# Image and Video Forensics

**DOI:** 10.3390/jimaging7110242

**Published:** 2021-11-17

**Authors:** Irene Amerini, Gianmarco Baldini, Francesco Leotta

**Affiliations:** 1 Department of Computer, Control and Management Engineering A. Ruberti, Sapienza University of Rome, 00185 Rome, Italy; leotta@diag.uniroma1.it; 2 European Commission, Joint Research Centre, 21027 Ispra, Italy; gianmarco.baldini@ec.europa.com

Nowadays, images and videos have become the main modalities of information being exchanged in everyday life, and their pervasiveness has led the image forensics community to question their reliability, integrity, confidentiality, and security more and more. Multimedia contents are generated in many different ways through the use of consumer electronics and high-quality digital imaging devices, such as smartphones, digital cameras, tablets, wearable sensors, and other Internet of Things (IoT) devices. The ever-increasing convenience of image acquisition has facilitated instant distribution and sharing of digital images on digital social platforms, determining a great amount of the exchanged data. Moreover, the pervasiveness of powerful image editing tools has allowed the manipulation of digital images for malicious or criminal ends, up to the creation of synthesized images and videos with the use of deep learning techniques. In all cases (e.g., forensic investigations, fake news debunking, information warfare, and mitigation of cyberattacks), where images and videos serve as critical demonstrative evidence, forensic technologies that help to determine the origin, authenticity of sources, and integrity of multimedia content become essential tools. In response to these needs, the multimedia forensics community has produced major research efforts regarding visual content authentication.

The call for papers for the Special Issue “Image and Video Forensics” was opened to anyone wishing to present advancements in state of the art, innovative research and ongoing projects on multimedia forensics and content verification to tackle new and serious challenges in ensuring media authenticity. This Special Issue solicited contributions from researchers in diverse areas such as image processing, artificial intelligence, computer vision and multimedia forensics.

This Special Issue received several submissions, which underwent a rigorous peer review process. After the review process, 18 articles (16 research papers and 2 review articles) were selected based on the ratings and comments. The published articles cover various applications of image and video forensics research, focusing on different branches such as forgery detection, deepfake detection, source identification and anomaly detection, and develop and apply a range of techniques, from image processing to computer vision, based on handcrafted features and/or deep learning.

The issue of media content authenticity verification has been taken into account from different points of view, considering traditional manipulation as well as more recent threats such as deepfakes. Rodriguez-Ortega et al. [1] presented a copy-move forgery detection technique based on a deep learning model to overcome the problem of generalization among different datasets. Alsakar et al. [2] focused instead on the analysis and identification of forgery in videos based on low computational complexity third-order tensor representation. Two types of forgery have been considered: insertion and deletion for static and dynamic videos. Ferreira S. et al. [3] exploited a support vector machine (SVM) classifier to distinguish between genuine and fake multimedia files, which may indicate the presence of deepfake content. This method was integrated as new modules in the widely used digital forensics application Autopsy. In their contribution, they proposed the extraction of a set of simple features resulting from the application of a discrete Fourier transform (DFT) to digital photos and video frames. Gardella et al. [4] focused on noise inconsistency in order to assess the authenticity of a digital image. To this end, they presented a multi-scale approach suitable for studying the highly correlated noise present in JPEG-compressed images. A noise level function was estimated for each image block and then compared with the noise level of the whole image. In the article proposed by Autherith and Pasquini [5], the detection of morphing attacks on digital faces was considered. To facilitate and improve this investigation they proposed the analysis of the locations of facial landmarks identified in two images, with the goal of capturing inconsistencies in facial geometry introduced by the morphing process.

Other contributions have addressed the problem of deepfake detection. Siegel et al. [6] tackled this issue, discussing if hand-crafted features could be used as an alternative to the learned features obtained through a deep learning algorithm. The proposed method made use of three sets of hand-crafted features and three different fusion strategies to implement DeepFake detection, demonstrating a similar generalization behavior to neural network-based methods.

Similarly to [6], Giudice et al. [7] focused their attention on deepfake image detection. To this end, they presented a new pipeline able to detect the GAN (generative adversarial network)-specific frequencies representing a unique fingerprint of the different generative architectures. By employing discrete cosine transformation (DCT), anomalous frequencies were detected and, in particular, the β statistics inferred by the AC coefficients distribution were used to recognize the different GAN engines that generated the data. Finally, Marcon et al. [8] addressed the problem of detecting manipulated videos of faces shared on social media platforms. In their contribution, a large scale performance evaluation was carried out involving general purpose deep networks and state-of-the-art manipulated data. The presented results confirmed that a performance drop was observed in every case where unseen shared data were tested by networks trained on non-shared data, finally concluding that fine-tuning operations can mitigate this problem.

Together with forgery detection, many challenging problems are faced by the multimedia forensics research community, such as source camera identification, the task of linking a particular digital image with its source device, social media identification, establishing the social network provenance of a certain image, as well as recovering from digital evidences the processing steps applied to the data, starting from the acquisition procedure up to tracking the spread and evolution of multiple images. De Roos and Geradts [9] investigated different factors, such as resolution, length of the video and compression, that influence camera video identification based on PRNU (photo response non-uniformity noise). To this end, Ferrara et al. [10] presented a new approach for the performance evaluation of source camera attribution by using likelihood ratio methods obtained from the PRNU similarity scores. Dal Cortivo et al. [11] investigated the camera model identification on video proposing a CNN (Convolutional Neural Network) based method jointly exploit audio and visual information. Ferreira A. et al. [12] focused their contribution on validating synthetic image detection and source linking methods on a new large scale dataset of printed documents.

The research community has recently shown an ambition to scale multimedia forensics analysis to real-world open systems. To this end, Maiano et al. [13] presented a method for assessing the social media platform of provenance of a video sequence, considering the interrelation among features captured from videos as well as those shared by images. Rouhi et al. [14] compared different classification-based methods to achieve both smartphone identification and user profile linking within social networks. 

Other contributions to this special session have addressed the problem of anomaly detection in unmanned aerial vehicle (UAV) video streams. Hamdi et al. [15] proposed an end-to-end architecture capable of generating optical flow images from original UAV images and extracting compact spatio-temporal characteristics for anomaly detection purposes. Karantaidis et al. [16] investigated the challenging problem of electric network frequency (ENF) estimation in static and non-static digital video recordings, designing an automated approach based on simple linear iterative clustering via the exploitation of areas with similar characteristics.

Finally, to conclude, two review articles have contributed to the success of this special issue. The first one is a comprehensive survey on anti-spoofing methods for facial recognition with Red Green Blue (RGB) cameras of generic consumer devices by Ming et al. [17]. The second one, by Castillo Camacho and Wang [18], covers the topic of deep learning-based methods for image forensics, reviewing methods dealing with forgery detection and source identification with an overview of adversarial forensics and of the main dataset used.
